# Seroprevalence of Human herpesvirus 8 (HHV-8) and incidence of Kaposi's sarcoma in Iran

**DOI:** 10.1186/1750-9378-6-5

**Published:** 2011-04-28

**Authors:** Somayeh Jalilvand, Zabihollah Shoja, Talat Mokhtari-Azad, Rakhshandeh Nategh, Ahmad Gharehbaghian

**Affiliations:** 1 School of Public Health, Tehran University of Medical Sciences, Tehran, Iran; 2 Research Center of Iranian Blood Transfusion Organization, Tehran, Iran

## Abstract

Seroepidemiological surveys show that the prevalence of human herpesvirus 8 (HHV-8) infection mostly varies in various geographical areas and reflects the local incidence of classic and endemic KS, being widespread in sub-Saharan Africa and Mediterranean countries and uncommon in the USA and Northern Europe. In the Middle East only few populations, such as Ashkenazi and Sephardic groups in Israel, have been adequately evaluated for HHV-8 seroprevalence. Among Iranian population a striking higher seroprevalence of HHV8 has been reported among haemodialysis (16.9%), renal transplant recipients (25%) and HIV (45.7%) patients compared to blood donors (2%). Kaposi's sarcoma (KS) is the rarest cancer in Iran, with an annual age-standardized incidence varying from 0.10 to 0.17 per 100,000 in males and from 0.06 to 0.08 per 100,000 in females. KS, however, is one of the most important malignancies in Iranian renal transplanted patients affecting up to 2.4% of organ recipients. The epidemiology of HHV8 and KS in Iran needs further evaluation. While the high prevalence of HHV-8 antibodies in HIV positive and haemodialysis individuals may be attributed to high-risk sexual behavior and polytransfusions, respectively, unknown determinants may be responsible for high seroprevalence of HHV8 and high incidence of KS in solid organ recipients. A global survey on HHV8 seroprevalence in Iran is mandatory to define co-factors associated with HHV8 infection and KS risk in the general Iranian population and in specific patient groups.

## Introduction

Kaposi's sarcoma (KS) is a mesenchymal tumor involving blood and lymphatic vessels that was first described in Eastern Europe in the late 19th century [1] and classically considered an indolent disease of elderly men. Now days, Kaposi's sarcoma has been classified in four different clinical and epidemiological forms [2]: 1) Classic KS, mainly occurring in elderly men of Mediterranean or Eastern European origin [3-6]; 2) African-endemic KS [6-9]; 3) Iatrogenic KS, developing in solid organ transplantation recipients [6,10-12] and 4) Epidemic or AIDS-associated KS [6,13-15]

In 1994 Chang et al. identified fragments of the Kaposi's sarcoma-associated herpesvirus (KSHV) genome in epidemic KS tissues, subsequently called human herpesvirus type 8 (HHV-8) [16]. HHV-8 is considered to be the etiological agent of all forms of Kaposi's sarcoma [2], and has been consistently associated with two types of lymphoproliferative disease, namely body cavity-based lymphoma [17] as well as multicentric Castelman's disease [16,18].

Seroepidemiological surveys have shown that HHV-8 infection is not ubiquitous [19]. The virus is less prevalent in northern Europe, North America, and most of Asia, and is more frequent in the Mediterranean area and parts of South America, and highly prevalent in sub-Saharan Africa [20-23]. Several studies have been performed in some Middle Eastern countries. In Israel seropositivity of HHV-8 has been ranging from 8.4% to 22% in healthy individuals [24,25]. In Saudi Arabia seroprevalence of HHV-8 was reported at 1.7% and 18% in healthy subjects and renal transplant recipients, respectively [26].

Few studies have been performed on the HHV-8 distribution and incidence of Kaposi's sarcoma in Iran. This study aimed to recapitulate available data on the seroepidemiology of HHV-8 and incidence of KS in the Iranian population. A systematic review of the published articles from January 1980 to December 2010 was conducted to assess the seroprevalence of HHV-8 and the incidence of KS in Iran. Data were identified by searches of Medline, Current Contents, PubMed, and references from relevant articles, with the search terms "Kaposi's sarcoma" or "HHV-8", and "Iran".

### Epidemiology of classic Kaposi's sarcoma in Iran

Data from the National Cancer Registry of Iran have reported that KS is the rarest cancer among Iranians [27]. Only 50, 44, and 61 KS cases were reported in the National Cancer Registry in 2004 [27,28], 2005 [27] and 2006 [27,28] respectively, and 101 new cases have been registered in Tehran Population Based Cancer Registry from 1998-2002 [27,29]. The annual age-standardized incidence rate was reported to be from 0.10 to 0.17 per 100,000 in males and from 0.06 to 0.08 per 100,000 in females [27]. Peak incidence has been reported at ages of 50-79 years. The male/female ratio in different reports varies from 3.2:1 to 1.8:1 and the elderly might be a common associated factor for KS [27]. There were no published reports about AIDS-KS in Iran.

Worldwide KS accounts for only 0.02% to 0.07% of all malignancies in the general population [30]. The regions with the highest incidence are Africa, where KS represents 3% to 9% of all cancer cases [31], Mediterranean and Eastern European areas, with specific geographic foci in Italy, Greece, and Israel [4,5].

### Epidemiology of post-transplant Kaposi's sarcoma in Iran

The incidence of KS in kidney recipients has increased following an enormous increase in the number of kidney transplantations during the recent decades, particularly of those of Mediterranean descent [32]. Post-transplantation KS develops in 23% to 28% of HHV8 seropositive patients and in only 0.7% of seronegative patients [32-34].

The rate of chronic kidney disease and renal transplantations has increased during the last two decades in Iran [34-36]. About 1.14-6% (mean = 2.8%) of Iranian renal transplant recipients develop cancer lesions, mostly skin cancers (Table 1) [37-43]. These findings are consistent with other report in Middle Eastern countries. The prevalence of all malignancies in renal transplant recipients was 1.9% in Pakistan [44], 4% in Turkey [45], 6.8% in Saudi Arabia [46], 4.8% in Kuwait [47], 5.9% in Iraq [48], and 1.7% in Jordan [49].

**Table 1:** List of published data on the incidence of Kaposi's sarcoma in renal transplanted patients in Iran

City	Renal Transplants	Male/Female (N)	Post-transplant Malignancies (%)	KS cases (%)	Male/Female (N)	Cutaneous/visceral (N)	Study period	References
Tehran	681	438/243	-	5 (0.73)	3/2	-	2000-2002	[52]
Tehran	100	53/47	6 (6)	2 (2.0)	2/0	-	2000-2002	[39]
Tehran	2211	-	-	10 (0.45)	8/2	8/2	1984-2007	[53]
Tehran	1750	-	28 (1.6)	13 (0.74)	-	-	1984-1999	[42]
Tehran	2050	-	-	18 (0.87)	13/5	18/1	1984-1999	[54]
Ahwaz	580	330/250	20 (3.4)	14 (2.4)	11/3	11/3	-	[40]
Babol	380	-	12 (3.15)	5 (1.3)	-	-	1999-2005	[41]
Shiraz	892	537/355	21 (2.3)	6 (0.68)	5/1	4/2	1988-2001	[38]
Tehran, Urmia, Babol, Sari, Tabriz, Hormozgan, Kerman	7,939	5018/2921	162 (2.04)	55 (0.69)	33/22	48/7	1984-2007	[37]
Tehran, Urmia, Babol, Sari, Tabriz, Hormozgan, Kerman, Isfahan, Ahwaz	11,255	7109/4146	128 (1.14)	77 (0.68)	48/29	-	1984-2008	[43]

KS is one of the most common cancers after renal transplantation in Iran [36] representing 28.5-70% (mean = 42.2%) of all post-transplant malignancies (Table 1) [37-43]. Other reports from the Middle Eastern region showed that KS was the most common post-transplantation malignancy with frequencies of 25% [45] and 68% in Turkey [50], 44% in Pakistan [51], and 50% in Iraq [48].

In Iran, incidence of KS among renal transplants varied from 0.45% to 2.4% in different studies (Table 1) [37,38,40-43,52-55]. In other countries of this region, the figures were 0.55% in Jordan [49], 3.2% in Turkey [56] and 4.9% in Saudi Arabia [46]. The incidence of KS was 0.7% [56], 1.7% [57] and 3.9% [58] among renal transplant recipients in Taiwan, Greece and South Africa, respectively. Therefore, the incidence of KS following kidney transplantation varies significantly in different geographic areas [59], and this is supporting the theory of ethnic or environmental factors in its pathogenesis. Globally, KS is most often seen in transplant recipients of Mediterranean, Jewish, Arabic, Caribbean, or African descent and the reported incidence ranges from 0.5% in most Western countries (including the United States) up to 5.3% in Saudi Arabia [32].

The KS incidence among Iranian transplants recipients had a peak during the first 2 years post transplantation. The time interval between transplantation and onset of KS was relatively early compared to other skin tumors. This observation is in agreement with other studies from the Middle East region reporting appearance of KS lesions in 6.5 to 27 months following kidney transplantation [26].

The mean-age of Iranian renal transplants developing KS is below 50 years, which is lower than that of patients with classic Kaposi's sarcoma (50-79 years). This finding is consistent with data reported by other studies on renal transplant patients from different regions of the world [30,32,45,60,61].

About 90% percent of transplant recipients affected by KS present cutaneous or mucosal lesions or both types. Visceral involvement occurred in 25% to 30% of kidney transplant patients [62]. Visceral lesions affected 10% of patients and predominantly involved the lymph nodes, gastrointestinal tract, and lungs [63]. In agreement with these worldwide studies, 80% of Iranian transplant recipients with KS developed cutaneous lesions. Visceral involvement was observed in 20% of patients (Table 1) [37,38,40,53,54].

Most cases of post-transplantation KS develop as a result of viral reactivation, since more than 80 percent of transplant recipients with KS are seropositive for HHV-8 before transplantation [34,64]. Renal recipient patients who were seropositive for HHV-8 before transplantation, have a risk to develop KS of 23% to 28% that is significantly higher compared to risk of 0.7% in patients who are seronegative before receiving a kidney transplant [32-34,65].

### Seroepidemiology of HHV-8 in Iran

Few serological survey on HHV-8 infection have been performed in Iran [55,66]. One study by Gharehbaghian et al. analyzed three groups of patients including haemodialysis patients, recruited in 2004 at the Immam-Khomeini hospital; HIV positive subjects, enrolled at the Immam-Khomeini hospital from July 2003 to June 2004; and blood donors, enrolled consecutively in a single day (June 28^th^, 2004) at the Research Center of Iranian Blood Transfusion Organization. The 256 blood donors, enrolled without any obligations, represented the 25.6% of blood samples screened on that day. All sera were tested for the presence of antibodies against HHV-8 lytic antigens by HHV-8 IgG EIA and by HHV-8 IgG IFA commercial kits (Biotrin, Ireland) as for manufacturer's instructions and each sample defined as "positive" if tested positive to both assays. The sensitivity of EIA and IFA tests was 90.4% and 100%, respectively, as reported in the manufacturer's catalogue. The specificity of EIA and IFA was 93% and 94%, respectively.

Among the 256 healthy Iranian blood donors (242 males and 14 females with mean age of 38 years, range 18-60 years) only 5 (2%, CI 95% = 0.003-0.03), including 4 males and one female, were positive for HHV-8 antibodies. Conversely, among the 118 haemodialysis patients (63 males and 53 females with mean age of 50 years), 20 (16.9%, CI 95% = 0.1-0.23) tested positive, including 8 males and 12 females. In the HIV positive group (33 males and 2 females, the range of age 20-40 years) 16 out of the 35 patients (45.7%, CI 95% = 0.29-0.62), all males, were positive for HHV-8 [55]. Also, frequency of HHV-8 positivity was not statistically significant different among haemodialysis patients stratified by number of transfusions (*P *= 0.36) [55]. Crude odds ratios and 95% confidence intervals were used to compare HHV-8 seroprevalence in different groups (Table 2). Overall, there was a statistically significant higher risk of HHV-8 seropositivity in haemodialysis (OR = 10.24, 95% CI: 3.5 - 32.1) and HIV (OR = 42.27, 95%CI: 12.7 - 150) patients compared to blood donors, although several variables between the three enrolled groups cannot be excluded.

**Table 2:** Seroprevalence of HHV-8 among blood donors, haemodialysis, HIV-positive and renal transplants Iranian patients

Study Population (N)	Male/Female (N)	HHV-8 positive cases (%)	HHV8 detection Methods	References
Blood donors (256)	242/14	5/256 (2%)	Lytic [IFA-EIA]*	[55]
Haemodialysis patients (118)	63/55	20/118 (16.9%)	Lytic [IFA-EIA]*	[55]
Renal transplants (100)	60/40	25/100 (25.0%)	LANA [IFA]**	[66]
HIV positive patients (35)	33/2	16/35 (45.7%)	Lytic [IFA-EIA]*	[55]

* Positivity was based on positive test of both EIA and IFA assays;

** Positivity was based on positivity to a single LANA IFA assay;

Low prevalence of HHV-8 in blood donors may indicate that virus is not widespread in this population; however, this study is not sufficient to determine the extrapolation of the true prevalence of HHV-8 in Iranian healthy population. Blood donors in above mentioned study were only from Tehran and it may be representative of the prevalence of HHV-8 in Tehran rather than Iran. The distribution of this virus probably is not ubiquitous in all over the Iran, so it is recommended to perform surveillance studies on different populations living in different areas of Iran in order to determine the global seroprevalence of HHV-8. Also blood donors commonly have not considered as persons with high risk behaviors. It is better that future study populations be general population rather than blood donors to estimate more relevant prevalence of HHV-8 infection.

In another study by Ahmadpoor et al., 100 serum from Iranian renal transplant recipients (60 male, 40 female) were analyzed for antibodies against the latent nuclear antigen of HHV-8 [66] and 25% tested seropositive for HHV-8 (Table 2). The mean age was 41.1 years (range, 17-74 years) and there was a statistically significant difference in HHV-8 seropositivity among recipients older than 55 years (*P *= 0.02). In particular, 8 out of 17 (47%) patients were seropositive in the group older than 55 years, versus 17 out of 83 (20%) patients in the group younger than 55 years. There were no significant differences in HHV-8 seropositivity regarding sex. Seropositive and seronegative patients were followed for 16 months and only one HHV-8 seropositive patient (1/25) developed Kaposi's sarcoma [66]. The risk of HHV8 infection in transplanted patients is significantly higher (OR = 16.73, 95% CI: 5.8 - 51.8) compared to blood donors, although different HHV8 detection methods were used (Table 2). These findings are consistent with other studies in our region reporting 18% and 28% HHV8 seroprevalence among renal transplant recipients in Saudi Arabia [26,67].

Different rates of HHV-8 infection have been reported in various populations in the world. The prevalence of HHV8 in healthy individuals was found to be 1.3%-4.4% in Southeast Asia and the Caribbean regions and > 40% in Africa [68-70]. In India a prevalence of 3.7% and 2.3% has been reported in healthy individuals and HIV positive patients, respectively [68]. In one study from Saudi Arabia the seroprevalence of HHV-8 in healthy Saudi national's people was reported to be 1.7% [26]. In Europe, the prevalence of HHV-8 was found to be lowest in Spain or Greece (6%-8%) and highest in Italy (20.4%) [71-75]. Approximately 50% of the adult population of Brazilian Amerindians was reported to have antibodies to HHV-8 [76], compared with only 11% of HIV-negative injection drug users in Argentina [77].

## Conclusions

In conclusion, in Iran a high prevalence of HHV8 infection has been observed in several risk groups such as haemodialysis, renal transplant and HIV-positive patients. KS incidence among renal transplant patients is as high as that observed in transplanted patients from endemic regions for KS. A future study including a large population from different regions of Iran is needed in order to define co-factors associated with HHV8 infection and KS risk specific patient groups in Iran.

## Competing interests

The authors declare that they have no competing interests.

## Authors' contributions

SJ conceived the study and performed a critical review of original data on HHV8 and KS prevalence in Iran in 1980-2010 period. ZS, TM-A and RN contributed to KS prevalence analysis in Iran. AG participated in the study design and coordination. All authors read and approved the final manuscript.
